# Impact of Amplification and Noise on Subjective Cognitive Effort and Fatigue in Older Adults with Hearing Loss

**DOI:** 10.3390/brainsci16020182

**Published:** 2026-01-31

**Authors:** Devan M. Lander, Christina M. Roup

**Affiliations:** Department of Speech and Hearing Science, The Ohio State University, Columbus, OH 43210, USA; roup.2@osu.edu

**Keywords:** hearing aids, effort, fatigue, cognitive performance, older adults

## Abstract

**Background/Objectives**: Older adults with hearing loss frequently report increased listening effort and fatigue, particularly in complex auditory environments. These subjective experiences may reflect increased cognitive resource allocation during both auditory and visual tasks, yet the impact of hearing aids on task-related effort and fatigue remains unclear. This study examined subjective effort and fatigue in experienced older adult hearing aid users while completing cognitively demanding auditory and visual tasks in quiet and background noise, with and without hearing aids. **Methods**: Thirty-one adults aged 60–87 years completed a cognitive battery assessing inhibition, attention, executive function, and auditory and visual working memory across four listening conditions: aided-quiet, unaided-quiet, aided-noise, and unaided-noise. Subjective effort was measured using the NASA Task Load Index, and task-related fatigue was assessed using a situational fatigue scale. Linear mixed-effects models controlled for age and pure-tone average hearing thresholds. **Results**: Participants reported significantly lower effort and fatigue in quiet compared to background noise, regardless of hearing aid use. The aided-quiet condition was rated as the least effortful and fatiguing, whereas the unaided-noise condition was rated as the most demanding. Subjective effort and fatigue were moderately to strongly correlated across conditions, particularly in noise. Auditory working memory performance was significantly associated with subjective fatigue across listening conditions, while visual working memory was not associated with effort or fatigue. Hearing aid use did not produce significant reductions in effort or fatigue across conditions. **Conclusions**: Background noise substantially increases perceived task-related effort and fatigue during cognitively demanding auditory and visual tasks in older adults with hearing loss. While hearing aids did not significantly reduce effort or fatigue across conditions, optimal listening environments were associated with the lowest subjective reports. Auditory working memory emerged as a key factor related to fatigue, highlighting the interplay between hearing, cognition, and subjective listening experiences in older adulthood.

## 1. Introduction

Older adults with hearing loss often report general complaints such as “At the end of the day, my hearing is worse” or “I have to focus a lot more at the end of the day to listen.” These experiences reflect the phenomena of subjective effort and fatigue, specifically in the domain of listening. Effort can be generally described as the purposeful allocation of resources to complete goal-oriented behavior [1]. Fatigue can result from the sustained effort put forth to participate in or complete a given task. Although a singular definition of fatigue does not exist, subjective fatigue can generally be described as a feeling of tiredness, exhaustion, or decreased motivation to continue an activity [2,3]. Older adults experience both cognitive decline and sensory decline (i.e., hearing loss), which may lead to greater sustained effort and more fatigue over time, ultimately resulting in a higher likelihood of withdrawal [4]. As a result, there are negative implications for social engagement and quality of life [5,6,7].

In addition to sensory decline, cognitive resources are known to decrease with age [8,9], impacting access to cognitive resources used for effort allocation. Reduced access to cognitive resources can potentially lead to fatigue more quickly, especially in cognitively demanding situations [10]. Older adults are likely to exert more effort and experience fatigue more quickly when compared to younger adults during cognitively demanding tasks [10]. The Framework for Understanding Effortful Listening model (FUEL Model) [11] conceptualizes the specific effects of listening on cognition and how this process is shaped by environmental conditions (e.g., background noise) and internal factors (e.g., age-related hearing loss). The availability of cognitive resources varies by individual; therefore, the point at which a task requires additional effort allocation will depend on the individual’s cognitive resources [12]. The FUEL model suggests that individual cognitive resources are allocated to tasks and, as task difficulty increases, more cognitive resources are required to complete the task, ultimately leading to fatigue [11,13,14].

Sensory decline in the form of hearing loss results in poorer access to the auditory signal, requiring more effort to process the incoming auditory signal and leading to listening-related fatigue. The primary intervention for age-related hearing loss to improve the auditory signal is hearing aids. A study by Monzani et al. [15] reported that hearing aids reduced effort and fatigue; however, a systematic review highlighted the mixed evidence regarding the impact of hearing aids on effort and fatigue [14]. In the large systematic review conducted by Ohlenforst and colleagues, the group concluded that across studies there was significant variability in methodology, and many studies were underpowered [14]. In addition, the authors pointed out the need for a common conceptual framework across studies. Thus, well-defined research is needed to clarify the potential benefit of hearing aids on subjective effort and fatigue across listening contexts.

Subjective effort and fatigue can be measured by a wide variety of measures. In hearing science, self-report instruments such as the NASA Task Load Index (NASA-TLX) [16,17,18] and the Vanderbilt Fatigue Scale for Adults (VFS-A) [19] have been used to quantify subjective experiences of listening effort and listening-related fatigue, respectively. These tools capture dimensions of workload, tiredness, and reduced motivation, making them potentially valuable for quantifying the task-related consequences of hearing loss, especially in challenging listening environments. By capturing perceived effort and fatigue, these measures provide insight into the cognitive load imposed by listening tasks, particularly for older adults with hearing loss.

The purpose of this project was to compare the subjective task-related effort and fatigue of experienced older adult hearing aid users while completing cognitive tasks in quiet and background noise. A unique feature of this study was that participants completed cognitive tasks where the target was both auditory and visual. A combination of auditory and visual cognitive tasks provides a more holistic view of the potential impact of hearing aids on effort and fatigue during cognitively demanding tasks. Overall, the study aimed to provide a greater understanding of how subjective effort and fatigue in older adults may change based on hearing aid use and the presence or absence of background noise.

## 2. Methods

### 2.1. Study Design

The present study was a cross-sectional, correlational research design. Data was collected from December 2023 to April 2024. The present study was approved by the Behavioral and Social Sciences Institutional Review Board. All participants provided written consent and were compensated for their time. The audiometric equipment used for the study was calibrated in accordance with the appropriate American National Standards Institute (ANSI) standards [20,21,22].

### 2.2. Participants and Testing Environment

Thirty-one adults 60–87 years of age were recruited to participate in the study. The mean age was 71.29 years old (SD = 6.85) with 17 females. Participants were recruited from the Department of Speech and Hearing Sciences Speech-Language-Hearing Clinic, the Ohio State University Campus, and the surrounding community of Columbus, Ohio. All participants underwent a cognitive screening using the Montreal Cognitive Assessment (MoCA) [23]. Only individuals with a score of ≥23 [24] were included. A visual screening for 20/40 vision was completed using a Snellen Eye Chart [25] with corrective lens (if needed) to ensure vision was not a barrier to completing the cognitive tasks on a computer screen. A visual screening for color blindness was conducted using the City University online Color Vision Test [26] to ensure color blindness would not interfere with the completion of the Stroop task, which requires color identification. Participants who failed the visual screenings were excluded. All participants were experienced hearing aid users (≥6 months) to ensure acclimatization to hearing aids. Participants completed a survey regarding hearing aid use to ensure regular usage (adapted from Bertoli et al., 2009) [27]. Individuals who reported <6 h per day and/or <5 days per week of hearing aid use were excluded from the study.

**Figure 1:**
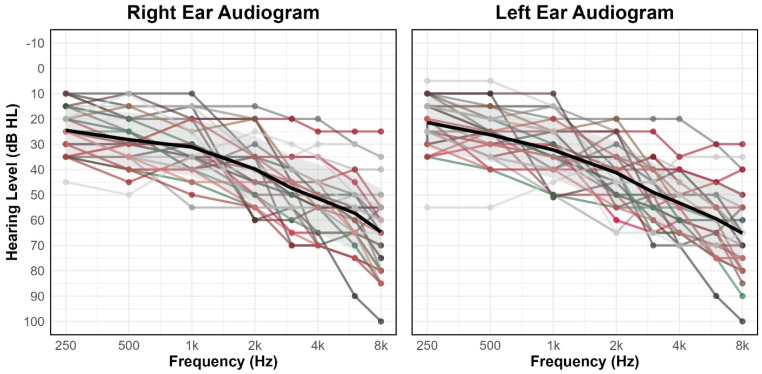
Individual audiograms for right and left ears with frequency (in kHz) on the abscissa and hearing level (in dB) on the ordinate. Thick black line represents the average audiogram for right and left ears. Shaded gray area represents one standard deviation from the mean.

Participants had symmetrical hearing (i.e., ≤15 dB HL ear difference) with no air–bone gaps exceeding 10 dB. All pure-tone thresholds 250–8000 Hz were present, with most participants presenting with a sloping mild to moderately severe high-frequency hearing loss consistent with presbycusis. Individual participant audiograms are displayed in Figure 1; the bolded black line represents the average, and the grey shading represents one standard deviation. Participants completed a questionnaire regarding health history and demographic information (e.g., age, socioeconomic status, education level). Individuals with a history of genetic hearing loss, chronic middle ear disease, ear surgery, meningitis, Ménière’s disease, stroke, and a history of ototoxic medication were excluded. All participants were native speakers of English. Further inclusion criteria were normal otoscopy and tympanometry [28]. Participants used a study hearing aid that was fit and verified to their hearing loss. All noise reduction and signal processing features were set to maximal settings for noise management.

Testing was completed in a double-walled sound booth. All auditory materials were presented through speakers via a sound card connected to the audiometer. All visual materials were presented on a laptop via MATLAB (Version R2022b) [29]. The order of conditions and tasks were counterbalanced across participants. Participants were tested in four conditions: in quiet and in background noise with and without hearing aids. Background noise was created using Adobe Audition [30] by mixing the Rainbow Passage [31] and cafeteria noise [32] at a 4 dB discourse-to-noise ratio (DNR).

### 2.3. Materials and Procedures

#### 2.3.1. Subjective Effort and Fatigue

The NASA-TLX [16] was used as a subjective measure of effort. The NASA-TLX assesses six domains post-task: mental demand, physical demand, temporal demand, perceived performance, effort, and frustration. The experimental tasks did not include a physical component, and therefore, for the purposes of this study, it was not included in the analysis. Table S1 includes the description of each domain and visualization of the analog scale. Participants rate each domain post-task on a visual analog scale which displays 10 large tick marks and 10 small tick marks. On all domains except performance, higher scores indicate the participant perceived a greater degree of the given domain (i.e., higher frustration = larger numerical score). Participants were instructed to mark anywhere on the scale to indicate their effort level for each domain of the NASA-TLX following each condition. Scores for each scale were calculated by domain from 0 to 100. An average effort score of the five domains (i.e., mental demand, temporal demand, perceived performance, effort, and frustration) was calculated for each participant in each experimental condition. The inverse of perceived performance score was used because a higher score on this scale suggests that the participant perceived better performance.

Participants completed a 22-item task-related fatigue scale (Table S2). Items on the scale were adapted from the VFS-A-40 [19] and were selected to address the level of fatigue following a specific task. Specifically, items were adapted from past to present tense. For example, “The tasks make me so tired I started to miss details.” versus “I get so tired from listening that I start to miss details in a conversation.” The questions addressed task-related fatigue as well as incorporating questions regarding listening. Ratings on the situational listening fatigue scale were a 5-point Likert scale (0 = not at all to 4 = extremely true). Lower scores on the scale indicate less task-related fatigue, and higher scores indicate more task-related fatigue. A total score out of 88 was calculated for each condition.

#### 2.3.2. Cognitive Test Battery

During each condition, the participants completed a cognitive battery which included three auditory and three visual tasks which measured inhibition, attention, executive function, and working memory. Table 1 provides an overview of each task.

#### 2.3.3. Research Design and Statistical Analysis

Statistical design was accomplished in consult with a statistician. Comparisons across conditions for measures of task-related effort and fatigue were analyzed using a linear mixed-effects model fit by REML [41,42]. Mixed-effects modeling is considered extremely useful in heterogeneous patient populations such as older adults with hearing loss due to the ability of the model to account for variability both within and across participants [42,43]. Correlation analyses were conducted between behavioral and subjective measures using IBM SPSS Statistics software version 29.0.1 [43]. For significant findings, a linear mixed-effects model was then used to explore relationships between the working memory tasks and task-related effort and fatigue (lme4) [44] to account for the repeated measures study design. All models were adjusted to account for both variability in age and pure-tone hearing status (i.e., PTA4). *p*-values were adjusted using the Tukey method. These analyses were conducted using Rstudio software (Version 4.4.0) [45]. One participant completed only part of the experiment due to an unforeseen illness that resulted in pressure equalizing tubes being placed bilaterally; the data for the condition that the participant did not complete were coded as missing for all statistical analyses.

## 3. Results

**Figure 2:**
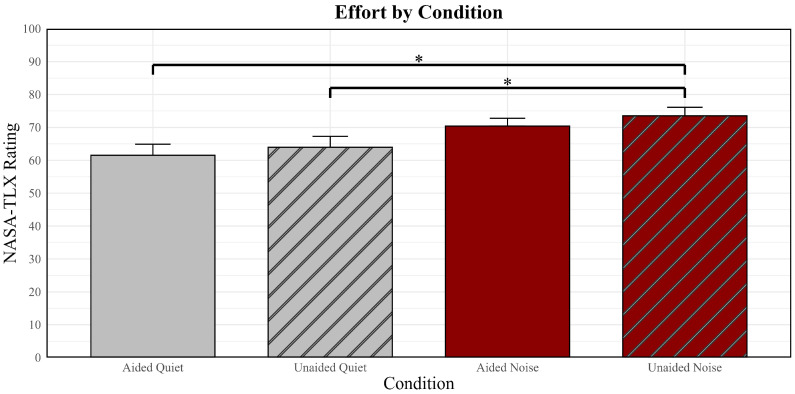
Mean reports of effort as measured by the NASA Task Load Index (NASA-TLX) and upward standard error bars. The conditions are represented on the abscissa, and effort ratings are represented on the ordinate. Asterisks indicate a significant difference between conditions.

**Figure 3:**
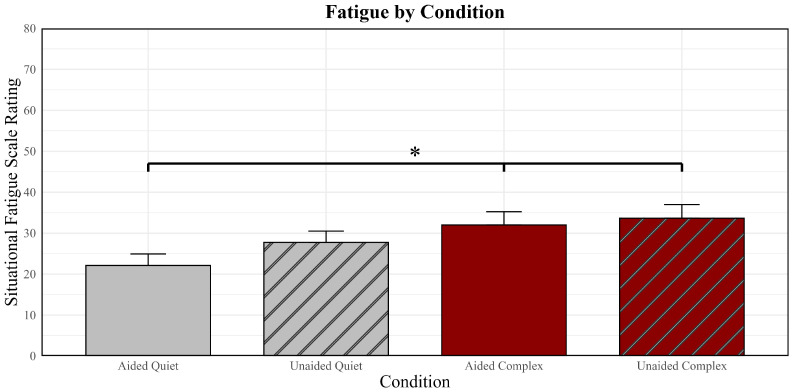
Mean reports of fatigue as measured by the situational fatigue scale and upward standard error bars. The conditions are represented on the abscissa, and fatigue ratings are represented on the ordinate. Asterisks indicate a significant difference between conditions.

Average listening effort and fatigue scores in quiet are presented in Figure 2 and Figure 3. As seen in Figure 2, older adult ratings of listening effort in quiet were similar when engaged in cognitive tasks with (aided $\;\bar{x}$ = 61.52) and without (unaided $\bar{x}$ = 63.97) hearing aids. In contrast, older adults rated cognitive tasks in quiet without hearing aids (unaided $\;\bar{x}$ = 27.74) as more fatiguing than cognitive tasks with hearing aids (aided $\;\bar{x}$ = 22.12).

Average listening effort and fatigue scores for the noise conditions are also presented in Figure 2 and Figure 3. Similar to the quiet condition, older adult ratings of listening effort in noise were similar when engaged in cognitive tasks with (aided $\;\bar{x}$ = 70.42) and without (unaided $\;\bar{x}$ = 73.53) hearing aids. Completion of cognitive tasks in noise also resulted in similar levels of fatigue with (aided $\;\bar{x}$ = 33.65) and without (unaided $\;\bar{x}$ = 32.00) hearing aids.

### 3.1. Comparing Effort and Fatigue in Quiet Versus in Noise

Differences in subjective listening effort between quiet and noise conditions were assessed with a linear mixed effects model controlling for age and pure-tone hearing. Table 2 presents the results of the model, including condition comparisons, standard errors, *t*-values, and *p*-values. Results revealed that the older adults rated the completion of the cognitive tasks in the quiet conditions (aided and unaided) as significantly less effortful than in the noise conditions (aided and unaided). The greatest difference in listening effort between conditions was observed when the older adults were completing the cognitive tasks in quiet with hearing aids (aided-quiet; easiest condition) versus in noise without hearing aids (unaided-noise; most difficult condition). Specifically, listening effort ratings were significantly greater in noise without hearing aids compared to in quiet with hearing aids. This suggests that quiet conditions while wearing hearing aids resulted in the least amount of listening effort when engaged in cognitive tasks.

Differences in subjective fatigue were also assessed between quiet and noise conditions with a linear mixed effects model controlling for age and pure-tone hearing. Results are also presented in Table 2. Similar to listening effort, results revealed that the older adults rated the completion of the cognitive tasks in the quiet conditions (aided and unaided) as significantly less fatiguing than in the noise conditions (aided and unaided). The difference in fatigue between conditions when the older adults were completing the cognitive tasks in quiet with hearing aids (aided-quiet; easiest condition) versus in noise without hearing aids (unaided-noise; most difficult condition) was significant (*p* = 0.002); however, comparing aided conditions was not significant (*p* > 0.05). Specifically, ratings of fatigue were significantly greater in noise without hearing aids compared to in quiet with hearing aids. This suggests that quiet conditions while wearing hearing aids resulted in the least amount of fatigue when engaged in cognitive tasks.

### 3.2. Correlations Between Effort and Fatigue

Partial Pearson correlations controlling for age and hearing loss (PTA4) were used for all correlation analyses. Ratings of listening effort and fatigue were significantly moderately correlated (r = 0.59, *p* = 0.001) without hearing aids in the quiet condition (unaided-quiet). Similarly, ratings of effort and fatigue were significantly correlated for both noise conditions (unaided-noise r = 0.73, *p* < 0.001; aided-noise r = 0.63, *p* < 0.001). In other words, older adults who reported greater degrees of listening effort also experienced greater levels of fatigue in quiet without hearing aids only and always in noise. Effort and fatigue ratings with hearing aids in the quiet condition (aided-quiet) were not correlated (r = 0.13, *p* = 0.51).

### 3.3. Correlations with Performance on Cognitive Measures

Partial correlations were analyzed to assess associations between task performance and perceived effort and fatigue in each condition. There were no correlations between performance on the trail-making task or the Stroop tasks and subjective ratings of effort and fatigue when accounting for age and PTA4. Relationships between effort, fatigue, and working memory task performance were further explored with linear mixed-effects models to account for participant variance and repeated measures methodology.

#### 3.3.1. Effort, Fatigue, and Visual Working Memory Task Performance

**Figure 4:**
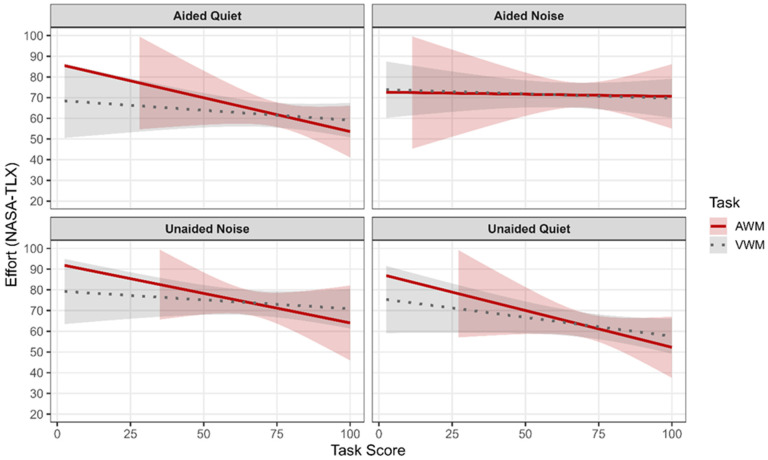
Predicted NASA-TLX ratings are plotted as a function of task performance (TaskScore) for auditory working memory (AWM) and visual working memory (VWM). The solid line represents AWM performance, and the dotted line represents VWM performance. Shaded regions indicate 95% confidence intervals for the predicted values. Each panel corresponds to a listening condition: Aided-Quiet, Aided-Noise, Unaided-Noise, and Unaided-Quiet. Predictions were derived from linear mixed-effects models controlling for participant age and pure-tone average (PTA4).

**Figure 5:**
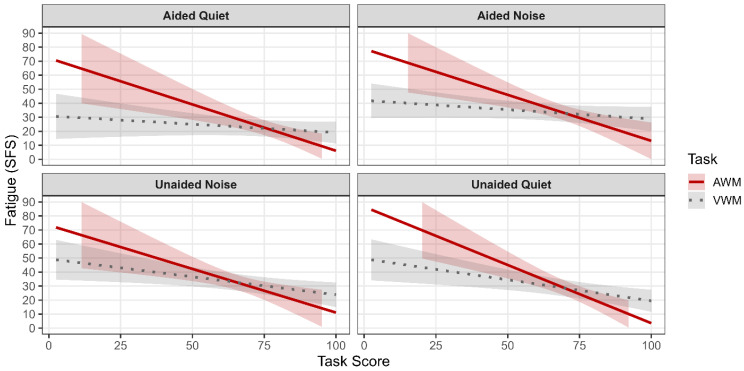
Predicted Subjective Fatigue Scale (SFS) scores are plotted as a function of task performance (TaskScore) for auditory working memory (AWM) and visual working memory (VWM). The solid line represents AWM performance, and the dotted line represents VWM performance. Shaded regions indicate 95% confidence intervals for the predicted values. Each panel corresponds to a listening condition: Aided-Quiet, Aided-Noise, Unaided-Noise, and Unaided-Quiet. Predictions were derived from linear mixed-effects models controlling for participant age and pure-tone average (PTA4).

Figure 4 and Figure 5 present model-predicted ratings of effort and fatigue across conditions as a function of task performance. The model demonstrated that NASA-TLX ratings were not significantly associated with SICSPAN scores (i.e., visual working memory) (β = −0.04, *p* = 0.68), and there were no significant interactions between SICSPAN performance and listening condition (*p*s > 0.24). Neither age nor PTA4 significantly predicted perceived effort (both *p*s > 0.42). Condition-specific slope estimates similarly indicated no reliable relationship between SICSPAN performance and effort in any listening condition.

Similarly, the model demonstrated performance on the SICSPAN was not a significant predictor of SFS scores (β = −0.13, *p* = 0.15). No significant interactions between SICSPAN performance and listening condition were observed (*p*s > 0.09). Age and PTA4 were not significant predictors of fatigue in the model (*p*s > 0.28).

#### 3.3.2. Effort, Fatigue, and Auditory Working Memory

The model demonstrated that NASA-TLX ratings were not significantly associated with WARRM scores (i.e., auditory working memory) (β = −0.02, *p* = 0.93), and there were no significant interactions between WARRM performance and listening condition (*p*s > 0.23). Neither age nor PTA4 significantly predicted perceived effort (*p*s > 0.31). Condition-specific slope estimates similarly indicated no reliable relationship between WARRM performance and effort in any listening condition.

In contrast, performance on the WARRM was a significant predictor of SFS scores (β = −0.66, *p* = 0.001), such that better auditory working memory performance was associated with lower reported fatigue across listening conditions. No significant interactions between WARRM performance and listening condition were observed (*p*s > 0.44), indicating that this relationship was consistent across aided and unaided conditions. Age was not a significant predictor of fatigue (*p* = 0.28), nor was PTA4 (*p* = 0.078).

## 4. Discussion

In daily life, hearing aid users are completing cognitively demanding visual and auditory tasks. It is not uncommon for older adults with hearing loss to report effort and fatigue, especially when completing a task in background noise. There is a limited body of research on listening effort and fatigue in older adults, and the development of reliable measures for these constructs is still in its early stages [3,46]. Some research has focused on and demonstrated that hearing aids decrease listening-related effort and fatigue [47,48]. However, it is unclear whether hearing aids decrease task-related effort and fatigue during cognitively demanding visual and auditory tasks in older adults. Examining the impact of hearing aids on subjective effort and fatigue during a cognitive battery of visual and auditory tasks provides a more holistic representation of task demands in the real world and the role that hearing aids play in this relationship. The current investigation compared ratings of subjective listening effort and fatigue from older adult hearing aid users following the completion of visual and auditory cognitive tasks in various conditions. The study also investigated the relationships between situational task-related effort, fatigue, and the performance on specific visual and auditory cognitive tasks of older adults.

### 4.1. Effort and Fatigue in Quiet Versus Noise

In the current study, older adult hearing aid users completed cognitively demanding tasks in four conditions: (1) with hearing aids in quiet (aided-quiet), (2) without hearing aids in quiet (unaided-quiet), (3) with hearing aids in background noise (aided-noise), and (4) without hearing aids in background noise (unaided-noise) conditions. After completing the behavioral tasks, participants rated their effort and fatigue for each condition. Altogether, older adults reported less effort and fatigue in quiet conditions compared to noisy conditions. Results revealed that completing tasks while using the hearing aids in quiet was significantly less effortful and fatiguing than listening with hearing aids in noise. These results are supported by previous literature. Specifically for listening-related tasks, Monzani et al. conducted a study in older adults investigating subjective effort and fatigue for older adult hearing aid users following an acclimatization period [15]. Monzani et al. concluded that hearing aid use significantly decreased the perception of effort and fatigue in their older adults, even when performance on listening tasks did not change. Similarly, in a study by Vaisberg et al. [48], participants completed a speech-in-noise task and rated their effort in aided and unaided conditions. Vaisberg and colleagues supported these subjective reports with functional near-infrared spectroscopy, which indicated that the brain regions associated with listening were less oxygenated, which suggests that participants were using fewer cognitive resources for listening when using hearing aids [48].

Other studies, particularly in the area of occupational health, have demonstrated that even for visual tasks, task-related effort and fatigue increase in the presence of background noise [49]. The current study confirmed that listening in noise significantly increased the perception of task-related effort and fatigue and expands on previous literature by including visual and auditory tasks. The impact of background noise on task-related effort and fatigue cannot be understated. As the auditory environment becomes more complex, there are less cognitive resources available to use for the task. Although in some cases performance on a task can be maintained, the consequence of background noise is increased effort and subsequently fatigue.

### 4.2. The Impact of Hearing Aids on Effort and Fatigue

The aided-quiet condition (i.e., most ideal) resulted in the lowest reports of task-related effort and fatigue. Ratings of effort were not significantly different between aided and unaided conditions in noise or quiet. Similarly, subjective fatigue was lowest in the aided-quiet (i.e., most ideal condition). However, the comparison across aided and unaided conditions was not significant. In other words, wearing hearing aids did not significantly decrease the reports of effort and fatigue between hearing aid conditions. This may be due to the level of effort required to complete the tasks across conditions. In other words, all conditions were effortful and fatiguing, or the results may be attributed to a relatively small sample size. In the case of both subjective effort and fatigue, the pattern of results suggests that participants perceived the aided-quiet condition as the easiest and the unaided-noise condition as the most difficult.

#### 4.2.1. Relationships Between Effort, Fatigue, and Cognitive Performance

As anticipated, subjective effort and fatigue were significantly correlated. This result confirms that individuals who report higher levels of effort also reports higher levels of subsequent fatigue. The relationship between effort and fatigue highlights the potential consequences of prolonged effort and subsequent fatigue. In a study conducted by DeBroux Leduc et al., focus groups were used to determine the factors that drive social isolation in older adults [50]. The results indicated that effort and subsequent fatigue was a consistent theme of their groups with older adults, indicating that if social situations are effortful and result in fatigue, they are less likely and motivated to return to that activity. This research supports that the management of sensory declines associated with older adulthood could be critical for older adults’ continued social participation. Research should continue to explore and define the relationships between effort and fatigue [14] and emphasize the treatment of sensory impairments such as hearing loss that result in increased cognitive demands when untreated. Recent results from the ACHIEVE study, a longitudinal study focused on the relationship between hearing aid use and cognition, suggest that hearing interventions are a low-risk strategy to promote continued social engagement for older adults [51]. Further exploration of the moderating relationships between hearing aid use, effort, fatigue, and outcomes for older adults may provide further clarification of the daily impact of hearing aid use on long-term outcomes for older adults.

#### 4.2.2. Associations with Cognitive Performance

Correlational analyses revealed that subjective fatigue was more frequently correlated with behavioral performance than effort. There were no significant correlations between listening effort and fatigue, measures of attention and executive function (i.e., trail-making task and oral trail-making task), and the visual measure of inhibition (i.e., visual and auditory Stroop task). One possible explanation for the lack of correlations in these tasks is that these measures were not particularly sensitive to measuring differences in attention, executive function, and visual inhibition in this task paradigm. A systematic review assessing papers exploring the relationship between hearing aid use and cognitive function emphasized a substantial need for standardization of well-justified cognitive measures [52]. A limitation of the current study is that although measures chosen were justified in other studies, in this case they did not result in significant results.

#### 4.2.3. Visual and Auditory Working Memory

Contrary to our initial hypotheses and prior correlational findings, the linear mixed-effects models indicated that visual working memory, as measured by the SICSPAN, was not significantly associated with subjective listening effort (NASA-TLX) or fatigue (SFS) across listening conditions. Neither main effects of visual working memory performance nor interactions with listening condition were observed for effort or fatigue outcomes. These results suggest that when age, hearing sensitivity, and within-subject variability across conditions are appropriately modeled, relationships between visual working memory, effort, and fatigue are not robust. The absence of significant relationships between SICSPAN performance and subjective effort contrasts with earlier analyses that suggested condition-specific associations in noise. One explanation is that such associations may reflect between-subject correlations that do not persist when repeated measures and covariates are accounted for in a mixed-effects framework. Ceiling and floor effects in the easiest condition (i.e., aided-quiet) and in the noise conditions (i.e., unaided-noise and aided-noise) respectively, may also have constrained variability in effort and fatigue ratings. The consistency of null findings across conditions indicates that visual working memory may play a limited role in subjective fatigue during visually based cognitive tasks, even in the presence of background noise.

In contrast, auditory working memory, as measured by the WARRM, demonstrated a distinct pattern of results. While WARRM performance was not significantly associated with subjective listening effort, it was a significant predictor of subjective fatigue across listening conditions. Specifically, poorer auditory working memory performance was associated with higher fatigue ratings, independent of listening condition, age, and hearing sensitivity. This finding suggests that auditory working memory capacity is closely linked to how cognitively fatiguing the tasks included in this study are perceived over time.

Importantly, the absence of significant interactions between auditory working memory and listening condition indicates that the relationship between WARRM performance and fatigue was stable across aided and unaided quiet and noisy conditions. This consistency implies that auditory working memory may reflect a more general cognitive resource related to sustained effort and mental endurance rather than condition-specific processing demands. It may also suggest that these specific subjective measures better predict auditory-related effort and fatigue.

These findings align with prior work demonstrating that older adults with stronger auditory working memory perform better in background noise [53,54,55] and report fewer subjective hearing difficulties [56]. Extending this literature, the current results suggest that auditory working memory may be particularly relevant for understanding subjective fatigue, even during tasks that are not explicitly auditory. This highlights the intersection of hearing loss, cognition, and individual perceptions of task demand.

The present results also complement qualitative findings reported by Davis et al., in which older adults described hearing aids as both alleviating and contributing to fatigue [57]. While hearing aids may reduce listening-related effort in some contexts, they may also increase cognitive load by amplifying irrelevant background sounds, particularly during visually demanding tasks without an auditory target. For individuals with poorer auditory working memory, this additional sensory input may exacerbate fatigue, even if perceived effort remains unchanged [46].

Together, these findings underscore the importance of auditory working memory as a key cognitive factor shaping subjective fatigue in older adults with hearing loss. Clinically, these results suggest that incorporating auditory working memory assessments may provide valuable insight into patients’ concerns regarding listening-related effort and fatigue. Future work should further disentangle how hearing aid processing, task modality, and cognitive capacity interact to influence effort and fatigue across real-world listening scenarios.

### 4.3. Limitations

Although the number of participants included in the study is consistent with other studies in the field (e.g., Blümer et al., 2024 [46]), the sample size does limit the ability to interpret the results. The within-subject design increases statistical efficiency by using each participant as their own control and reduces between-person variance. Further, linear mixed-effects models account for repeated measurement and leverage all observations, making them ideal for heterogeneous populations [42,43].

A methodological limitation of this study is that the ratings of effort and fatigue were not divided by visual and auditory tasks. Had participants rated their effort and fatigue for visual and auditory tasks independently, the impact of hearing aids on subjective fatigue and effort for visual and cognitive tasks may have been clearer. Future studies should consider investigating the impact of hearing aids on effort and fatigue by grouping tasks by visual and auditory domain rather than only by listening condition. Another clear limitation of this study is the ecological validity of the cognitive tasks. Tasks were conducted in a laboratory environment and did not mirror everyday cognitive tasks, and therefore, the results should be interpreted with caution.

## 5. Conclusions

In summary, the findings from the current study provide a novel perspective to investigate the relationships between hearing aids, effort, fatigue, and background noise in older adults with hearing loss. A key takeaway from this study may be that the results indicate that background noise can impact the perception of effort and fatigue for both auditory and visual cognitive tasks. Further investigation will be imperative to clarify the multifaceted nature of subjective effort and fatigue of older adults with hearing loss in complex listening environments.

## Figures and Tables

**Table 1:** Description of cognitive tasks included in the battery.

Task	Type	Description
Auditory Stroop Task [33,34]	Auditory	Uses male and female voices to present the words “mother,” “father,” and “person” with speaker in the sound field. Congruent and incongruent trials are presented, and the participant must determine if the speaker is male or female.
Stroop Color Word Task [35,36]	Visual	A series of color words are presented on a computer screen. Congruent and incongruent trials are presented, and the participant must determine the color of the font.
Oral Trail Making Test [37,38]	Auditory	Included part A and B. Part A required participants to count from 1–25. Part B required participants to alternate letters and numbers in ascending order (i.e., A-1, B-2, C-3, etc.). Participants were required to auditorily monitor their responses for accuracy. When an error was made, participants were reoriented to the correct response.
Trail Making Test [37,38,39]	Visual	On a computer screen, participants connected letters and numbers in ascending order (i.e., A-1, B-2, C-3, etc.). Only correct responses allowed participants to move forward with the task.
Word Auditory Recognition and Recall Measure [40]	Auditory	A sound field speaker is used to present the target words. Participants listen, repeat, and categorize the word in either the first (A–M) or second (N–Z) half of the alphabet. Words are further organized into sets starting at two and increasing to five words. Participants complete five trials of each set size. After listening, repeating, and categorizing each word in the set, the participant is prompted to repeat all the words in the set.
Size Comparison Span Test [35]	Visual	The task presents comparison words (e.g., “Is an ANT smaller than a CAT?”) followed by the presentation of a to-be-remembered word. The participant must identify if the statement is true or false while also keeping track of the to-be-remembered words. For this task, set sizes began at two words and increased to six words. Each set size was presented twice. Participants were required to recall the to-be-remembered words following each set.

**Table 2:** Estimates represent model-adjusted (i.e., controlling for age and pure-tone average [PTA4]) mean differences in subjective effort and fatigue scores between conditions. Positive estimates indicate higher ratings for the first condition listed. Linear mixed-effects models included random intercepts for participants. *p* values reflect Tukey-adjusted pairwise comparisons.

	Effort				Fatigue			
Contrasted Conditions	Estimate	SE	*t*	*p*	Estimate	SE	*t*	*p*
Aided Noise–Aided Quiet	9.74	3.66	2.66	0.066	11.68	2.59	4.50	<0.001 **
Aided Noise–Unaided Noise	−2.47	3.70	−0.67	0.963	2.00	2.62	0.76	0.872
Aided Noise–Unaided Quiet	8.65	3.66	2.36	0.134	6.03	2.59	2.33	0.100
Aided Quiet–Unaided Noise	−12.21	3.70	−3.30	0.011 *	−9.68	2.62	−3.69	0.002 **
Aided Quiet–Unaided Quiet	−1.10	3.66	−0.30	0.998	−5.65	2.59	−2.18	0.138
Unaided Noise–Unaided Noise	11.12	3.70	3.01	0.026 *	4.04	2.62	1.54	0.418

Note: SE = standard error; *t* = *t*-value, *p* = *p*-value. (*) represents *p*-values < 0.05 and (**) represent *p*-values < 0.01.

## Data Availability

The original data supporting the conclusions of this article will be made available by the authors on request. Data is private as it is being used for ongoing research.

## Supplementary Materials

The following supporting information can be downloaded at: https://www.mdpi.com/article/10.3390/brainsci16020182/s1, Table S1: NASA-TLX; Table S2: Situational Fatigue Scale.

## Abbreviations

The following abbreviations are used in this manuscript:

FUEL	Framework of Effortful
NASA-TLX	NASA Task Load Index
VFS-A	Vanderbilt Fatigue Scale for Adults
PTA4	Four Frequency Pure-tone Average
SFS	Situational Fatigue Scale
SICSPAN	Size-Comparison Test
WARRM	Word Auditory Recognition and Recall Measure
