# Supplementary material for: Impact of Amplification and Noise on Subjective Cognitive Effort and Fatigue in Older Adults with Hearing Loss
Source: Brain Sci. 2026 Jan 31;16(2):182. doi: 10.3390/brainsci16020182 (PMC12938605; doi:10.3390/brainsci16020182)
Supplement: Supplementary file 1 [file brainsci-16-00182-s001.zip › brainsci-4082071-supplementary.pdf]

Table S1. NASA-TLX. The table contains the domains and domain description prompt from the NASA-TLX. The final row is a sample of visual analog scale used for each domain.

**Domain**	**Prompt**
Mental Demand	How much mental demand and perceptual activity was required (e.g., thinking, deciding, calculating, remembering, looking, searching, etc.)? Was the mission easy or demanding, simple or complex, exacting or forgiving?
Temporal Demand	How much time pressure did you feel due to the rate or pace at which the mission occurred? Was the pace slow and leisurely or rapid and frantic?
Performance	How successful do you think you were at accomplishing the goals of the mission? How satisfied were you with your performance in accomplishing these goals?
Effort	How hard did you have to work (mentally and physically) to accomplish you level of performance?
Frustration	How discouraged, stressed, irritated, and annoyed versus gratified, relaxed, content, and complacent did you feel during your mission?

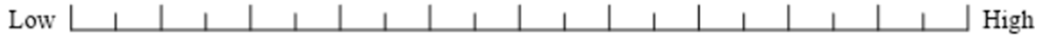

Table S2. Situational Fatigue Scale.

	Item	Not at all	Slightly True	True	Mostly True	Extremely True
1	It took A LOT of mental energy to complete the task.	0	1	2	3	4
2	I became mentally tired following the task.	0	1	2	3	4
3	The task required a lot of energy to complete.	0	1	2	3	4
4	I feel worn out from the task.	0	1	2	3	4
5	I need a nap after completing this task.	0	1	2	3	4
6	If given the option, I would have chosen to end the tasks early.	0	1	2	3	4
7	I was frustrated by trying to listen.	0	1	2	3	4
8	I am exhausted from listening during the tasks.	0	1	2	3	4
9	I feel emotionally tired due to trouble hearing and understanding.	0	1	2	3	4
10	The tasks make me so tired I started to miss details.	0	1	2	3	4
11	This activity gave me a headache	0	1	2	3	4
12	I will go to bed early after completing this activity.	0	1	2	3	4
13	I will rearrange my day to include rest after completing this activity.	0	1	2	3	4
14	I feel emotionally drained after this task.	0	1	2	3	4
15	I will avoid noisy situations for the rest of the day after completing this task.	0	1	2	3	4
16	This task was stressful.	0	1	2	3	4
17	I am mentally tired.	0	1	2	3	4
18	I am tired from trying to listen and understand.	0	1	2	3	4
19	I notice I feel fatigued in similar challenging listening situations.	0	1	2	3	4
20	These tasks were mentally taxing for me.	0	1	2	3	4
21	I will not socialize following this activity due to tiredness.	0	1	2	3	4
22	I prefer to avoid social events following similar activities.	0	1	2	3	4
